# Transcription Inhibition by DRB Potentiates Recombinational Repair of UV Lesions in Mammalian Cells

**DOI:** 10.1371/journal.pone.0019492

**Published:** 2011-05-05

**Authors:** Ivaylo Stoimenov, Niklas Schultz, Ponnari Gottipati, Thomas Helleday

**Affiliations:** 1 Department of Genetics, Microbiology, and Toxicology, Stockholm University, Stockholm, Sweden; 2 Gray Institute for Radiation Oncology and Biology, University of Oxford, Oxford, United Kingdom; University of Minnesota, United States of America

## Abstract

Homologous recombination (HR) is intricately associated with replication, transcription and DNA repair in all organisms studied. However, the interplay between all these processes occurring simultaneously on the same DNA molecule is still poorly understood. Here, we study the interplay between transcription and HR during ultraviolet light (UV)-induced DNA damage in mammalian cells. Our results show that inhibition of transcription with 5,6-dichloro-1-beta-D-ribofuranosylbenzimidazole (DRB) increases the number of UV-induced DNA lesions (γH2AX, 53BP1 foci formation), which correlates with a decrease in the survival of wild type or nucleotide excision repair defective cells. Furthermore, we observe an increase in RAD51 foci formation, suggesting HR is triggered in response to an increase in UV-induced DSBs, while inhibiting transcription. Unexpectedly, we observe that DRB fails to sensitise HR defective cells to UV treatment. Thus, increased RAD51 foci formation correlates with increased cell death, suggesting the existence of a futile HR repair of UV-induced DSBs which is linked to transcription inhibition.

## Introduction

There is a need to coordinate transcription, replication and DNA repair occurring simultaneously on the same DNA molecule. Homologous recombination (HR) is intricately associated with replication [1], [2], transcription [3], [4] and DNA repair [5] in all organisms studied. Loss of HR results in accumulation of translocations, deletions and DNA double-strand breaks (DSBs), which can promote cancer development as in the case of BRCA1 or BRCA2 defective tumours [6]. The levels of HR are elevated in actively transcribed regions, which is likely related to DNA replication forks that need to bypass RNA polymerase [4], [7], [8], [9]. There is evidence that inhibition of transcription increases recombination levels in yeast [10], [11] and mammalian cells [12], which can be explained by impaired replication fork progression [4], [13].

One possible situation, where HR and transcription compete for the same substrate is in the repair of UV lesions. UV damage is largely repaired by nucleotide excision repair (NER), although HR is also involved [14]. There is evidence that exposure to UV radiation increases HR [15]. Furthermore, it is well established that UV damage occurring in actively transcribed DNA is repaired more quickly, in a process called transcription-coupled repair (TCR) [16]. This places the transcription machinery at the very core of the damage recognition and subsequent repair of UV-induced DNA lesions in actively transcribed chromatin. There is a process of transcription-associated recombination (TAR), which directly links recombination and transcription in both lower and higher eukaryotes [17].

Here, we report that transcription inhibition induces HR repair of UV lesions. In our work we use 5,6-dichloro-1-beta-D-ribofuranosylbenzimidazole (DRB) as a transcription inhibitor. This compound prevents activating phosphorylations of the RNA polymerase II C-terminal domain (CTD) [18], [19], which results in repression of transcription elongation [19] and dramatic reduction in mRNA levels [20]. Our results show that inhibition of transcription with DRB potentiates UV-induced DNA DSBs, toxicity and HR. Since DRB does not potentiate UV-induced toxicity in HR defective cells, we suggest that the induction of HR mediates the increased UV-induced toxicity by DRB. We speculate that this is related to a futile HR repair of DSBs occurring in the absence of a sister chromatid.

## Materials and Methods

### Cell culture

The Chinese hamster ovary derivative cell lines AA8, irs1SF and UV5 used in this study were all obtained from Dr. Larry H. Thomson (LLNL, Livermore, CA) [21], [22]. All cell lines were cultured in Dulbecco's Minimum Essential Medium (DMEM) purchased from GIBCO, supplemented with 10% foetal bovine serum (GIBCO, E.U. Approved, South American source) and 90 U.ml^−1^ of penicillin-streptomycin (Invitrogen). For synchronization experiments, serum was temporary reduced to 0.1% for 48 h prior the treatments applied.

### Clonogenic survival assay

Cells were plated overnight in 100 mm Petri dishes, rinsed once with PBS and treated with UV irradiation in a minimal amount of PBS. The UVC irradiation is administrated from a low pressure mercury lamp (Phillips TUV 15W) at 254 nm and a rate of 0.18 J.m^−2^.sec^−1^. The exposure time was controlled using a fast magnetic shutter, mounted within the apparatus. After the UV irradiation the cells were left in DMEM in the absence or presence of 20 µM DRB (Sigma-Aldrich Co.) or other transcription inhibitors: actinomycin D, α-amanitin or flavopiridol (all from Sigma-Aldrich Co.) for 24 h. The dishes were then washed twice with PBS and supplemented with fresh DMEM. Seven to ten days later, when colonies could be observed, they were fixed and stained with methylene blue in methanol (4 g.l^−1^).

### Immunofluorescence

Cells were plated onto cover slips, allowed to attach for 24–48 h and grown for 24 h in the presence or absence of treatments as indicated. Cover slips were rinsed in phosphate-buffered saline (PBS) and fixed in 3.7% paraformaldehyde, 0.1% Triton X-100 for 20 min at room temperature. Cover slips were extensively washed (PBS, 0.3% Triton X-100 for 10 min and 3×5 min in PBS, 0.05% tween20) before blocking in 3% BSA for 1 h and thereafter incubated with primary antibody for 20 h at 4°C. The cover slips were washed as above followed by 1 h incubation at room temperature with the appropriate secondary antibody and washed again as above. Cover slips were washed in PBS, DNA stained with 1 µg/ml To-Pro-3 (Molecular Probes) and mounted in Pro Long Gold (Molecular Probes).

Primary antibodies used were rabbit polyclonal RAD51 (H-92, Santa Cruz 1∶1000) and mouse monoclonal anti-γH2AX (clone JBW301, Millipore 1∶1000). The secondary antibodies were AlexaFluor 555 donkey anti-rabbit IgG (Molecular Probes), AlexaFluor 488 donkey anti-mouse IgG (Molecular Probes). Antibodies were diluted 1∶500 in PBS containing 3% BSA and 0.05% tween20.

Images were obtained with a Zeiss LSM 510 inverted confocal microscope using a planapochromat 63x/NA 1.4 oil immersion objective. Through focus maximum projection images were acquired from optical sections 0.50 µm apart and with a section thickness of 1.0 µm. Images were processed using Adobe Photoshop or Adobe Illustrator (Adobe Systems Inc). The frequencies of cells containing foci were determined in six experiments. At least 200 nuclei were counted on each slide.

### Detection of BrdU incorporation by FACS

Exponentially growing or serum starved cells were labelled with 10 µM BrdU for 45 min, after which the cells were harvested by brief trypsinisation and washed in PBS. They were fixed with ice-cold 80% ethanol and stored overnight at +4°C. After PBS wash, the cells were treated with freshly made 2M HCl for 30 min at room temperature. After three washes with PBS, the pellet was incubated with anti-BrdU antibody at a dilution of 1∶100 for 1 h at room temperature. After two subsequent washes with PBS-T (PBS, 0.1% Triton X-100), the secondary antibody (donkey anti-mouse Alexa Fluor 488) was added at a dilution of 1∶200 for 1 h at room temperature. After a PBS wash, the material was treated with 25 µg/ml Propidium Iodide and 100 µg/ml RNAse A (Invitrogen) and for at least 30 min. The cell cycle profile was analyzed by a FACSCalibur flow cytometer. Data from 20 000 events were collected.

## Results

### DRB treatment increases UV sensitivity, but not in HR defective cells

**Figure 1:**
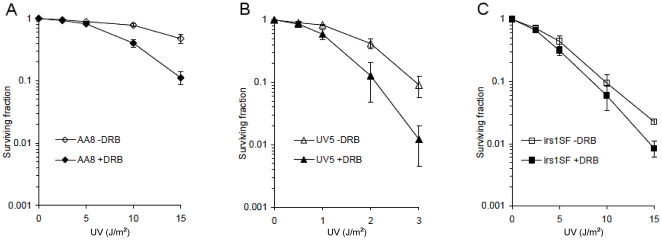
Transcription inhibition increases UV sensitivity, but not in HR defective cells. Clonogenic survival assay was performed in AA8 (**A**), UV5 (**B**) and irs1SF (**C**) cell lines after treatment with increasing doses of UV. In the case of the UV5 cell line (**B**), the doses of UV were considerably lower, since the cell line is highly sensitive to UV-irradiation. The experiment compares control cells to cells treated with DRB for 24 h post UV irradiation. The graphs depict the mean and standard deviation of at least three independent experiments.

It is well established that repair of UV-induced lesions is much more rapid in transcribed DNA, in a process called transcription-coupled repair (TCR) [16]. Here, we treated wild type Chinese hamster ovary cells (AA8) with increasing doses of UV in the presence or absence of the transcription inhibitor DRB. We found that DRB sensitises wild type cells to UV treatments (Figure 1A), which is unsurprising as inhibition of transcription prevents TCR of UV lesions [23]. We then treated UV5 cells, defective in XPD (ERCC2) [22], with UV in the presence or absence of DRB. XPD is a helicase and part of the transcription factor IIH (TFIIH). It is required for NER, both for TCR and global-genome repair (GGR) [24]. We confirm that UV5 cells are highly sensitive to UV lesions (Figure 1B). Inhibition of transcription in TCR defective cells would be expected to have no further effect on UV-sensitivity, although we cannot rule out the possibility that transcription of a factor needed for UV survival is required. We found that UV5 cells maintained increased sensitivity to DRB, similar to wild type cells, which is unexpected given that UV5 cells are already defective in TCR. Thus, the data suggest that there may be a mechanism promoting survival, which requires transcription and is NER independent.

We then treated XRCC3 mutated and HR defective irs1SF cells [21] with UV in combination with DRB. We confirm the UV sensitivity of irs1SF cells [14], implying a role of HR in the repair of UV damage. In *E. coli*, it has been shown that HR is involved in post replication repair of UV-induced DNA gaps [25], which is also believed to be the case in human cells. Here, we find that co-treating irs1SF cells with DRB did not increase their sensitivity to UV (Figure 1C). This provides genetic evidence that the increased sensitivity to UV by transcription inhibition observed in wild type and NER defective cells is mediated by HR.

Next we wanted to test whether other transcription inhibitors increase sensitivity to UV treatment. We performed a clonogenic survival assay in AA8 and irs1SF cells, treated with actinomycin D, α-amanitin or flavopiridol (Figure S1). Similarly to DRB, all the transcription inhibitors tested increased sensitivity of AA8 cells to UV treatment at the higher concentrations. Irs1SF cells displayed weak or no increase in sensitivity (Figure S1).

**Figure 2:**
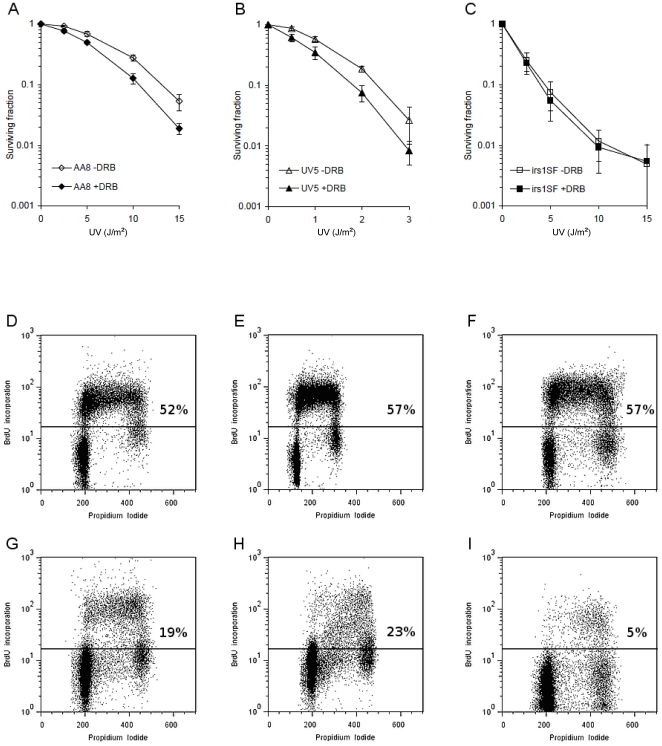
The effect of transcription inhibition on UV sensitivity is decreased after serum starvation. Clonogenic survival assay in AA8 (**A**), UV5 (**B**) and irs1SF (**C**) cell lines after synchronization by serum starvation. The cells were serum starved for 48 h and then treated with increasing doses of UV, incubated in the presence or absence of DRB for 24 h and cultivated in fresh medium. In the case of the UV5 cell line (**B**), the doses of UV were considerably lower, since the cell line is highly sensitive to UV-irradiation. The graphs depict the mean and standard deviation of at least three independent experiments. Flow cytometry plots of unsynchronized (upper row, AA8 (**D**), UV5 (**E**) and irs1SF (**F**)) and synchronized (lower row, AA8 (**G**), UV5 (**H**) and irs1SF (**I**)) cells, showing reduction in S-phase population after serum starvation. BrdU incorporation was used as a marker of ongoing replication. S-phase cut-off is shown as a percentage of total number of cells.

In mammalian cells, HR is primarily involved in repair of replication-associated lesions [2], [26] and the success of HR repair is dependent on the presence of a sister chromatid [27], [28]. Since the DRB potentiated UV-induced toxicity requires functional HR, we wanted to test if the DRB potentiation is stronger in replicating cells, when a sister chromatid is present. To test this, we UV treated serum starved G1-arrested cells in the presence or absence of DRB. Although, the magnitude of the UV-sensitising effect of DRB decreases in G1 arrested cells (Figure 2), the relative DRB sensitisations remain similar as compared to exponentially growing cells (Figure S2). Furthermore, we observed that the treatment was in general more toxic to the cells after synchronisation, which is similar to the effects seen after ionizing radiation [29] and likely explained by additional repair pathways available in the replicating cells (i.e. HR repair).

### DRB treatment potentiates UV-induced DNA damage

**Figure 3:**
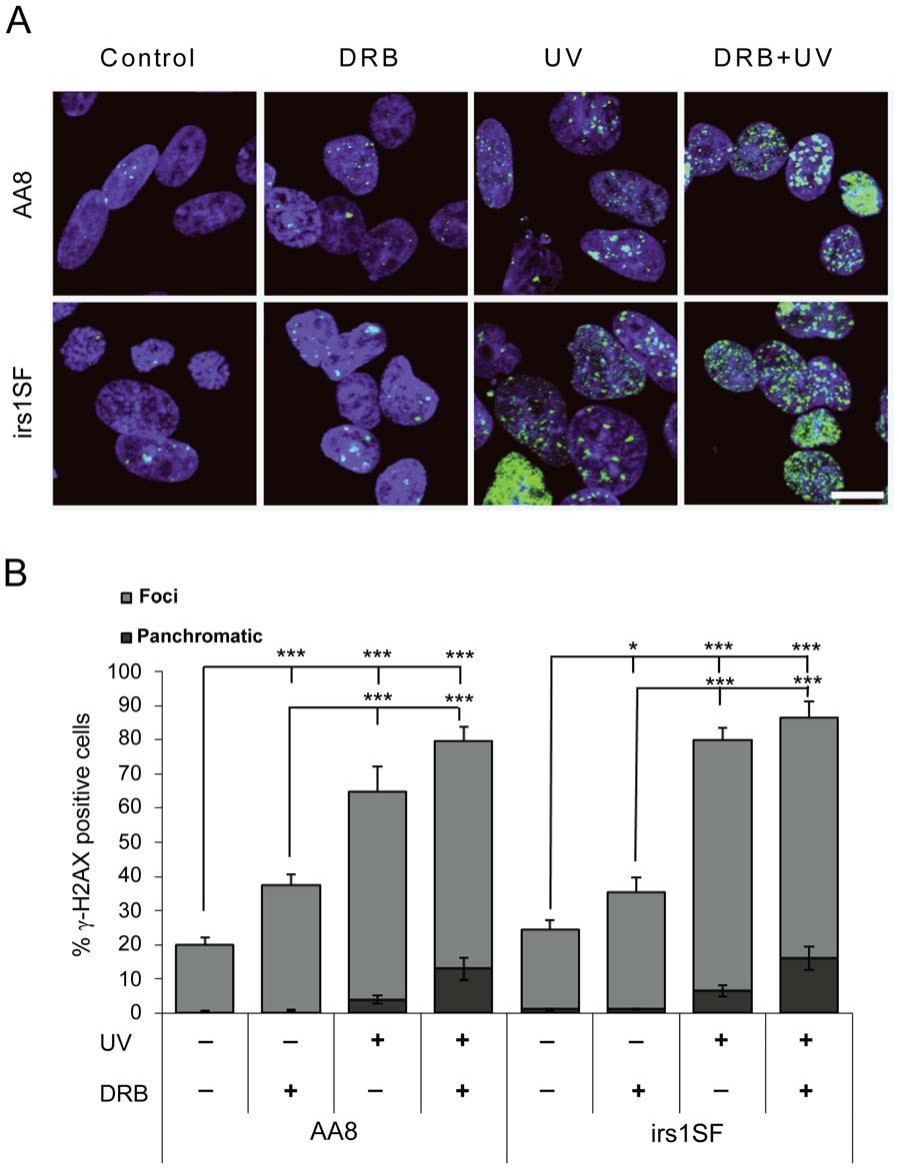
UV exposure and transcription inhibition induce γH2AX foci formation in mammalian cells. AA8 and irs1SF cells were treated with 20 µM DRB for 24 h, 10 J.m^−2^ UV or both. (**A**) Confocal through focus maximum projection images showing γH2AX staining in green and DNA staining in blue. Bar 10 µm. (**B**) Quantification of γH2AX positive cells. Cells with more than 10 bright foci were considered positive. Strong and evenly stained nuclei were classified as panchromatic. The means and S.E. (bars) of six experiments with 200 cells counted for each experiment are shown. Values marked with asterisks are significantly different from control in T-test (*P<0.1, ***P<0.001).

**Figure 4:**
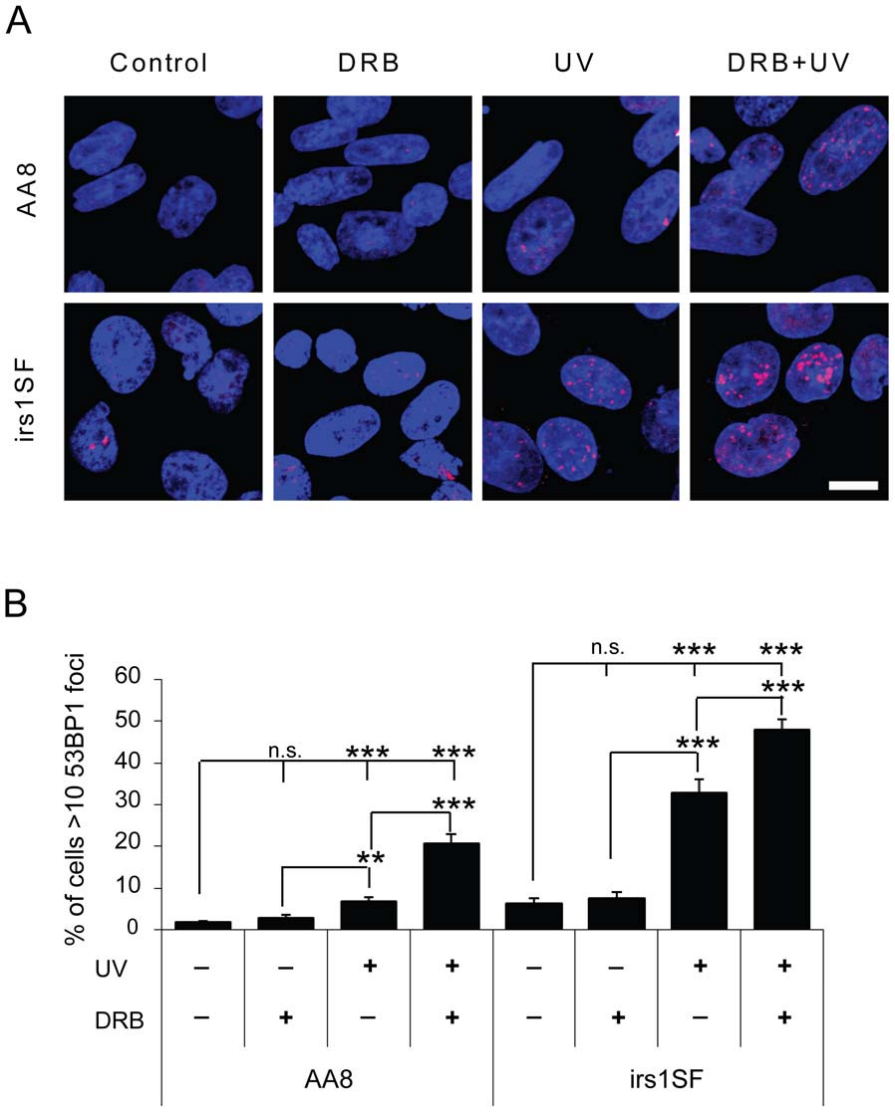
DRB potentiates the formation of DSBs by UV treatment, as seen by an increase in 53BP1 foci formation. AA8 and irs1SF cells were treated with 20 µM DRB for 24 h, 10 J.m^−2^ UV or both. (**A**) Confocal through focus maximum projection images showing 53BP1 staining in red and DNA staining in blue. Bar 10 µm. (**B**) Quantification of 53BP1 positive cells. Cells with more than 10 bright foci were considered positive. The means and S.E. (bars) of six experiments with 200 cells counted for each experiment are shown. Values marked with asterisks are significantly different from control in T-test (**P<0.01, ***P<0.001).

To test the underlying mechanisms for DRB dependent UV sensitisation we determined the amount of γH2AX foci in a series of experiments using AA8 and irs1SF cells, treated with UV and/or DRB. We found that UV-induced DNA damage is increased with the addition of DRB and is unrelated to HR proficiency (Figure 3). This suggests that DRB treatment itself induces DNA damage. Formation of γH2AX foci is commonly used as a marker for DNA DSBs [30], [31]. However, there are ongoing discussions as to the specificity of γH2AX as a clear indication of DSBs [30], [32]. To confirm that DRB indeed causes the formation of DSBs, we followed the formation of 53BP1 foci as a marker of DSBs [30]. We confirm that DRB potentiates the formation of UV-induced DSBs, as observed by an increase in 53BP1 foci, particularly in the HR defective irs1SF cells (Figure 4).

### DRB treatment potentiates the formation of UV-induced RAD51 foci

**Figure 5:**
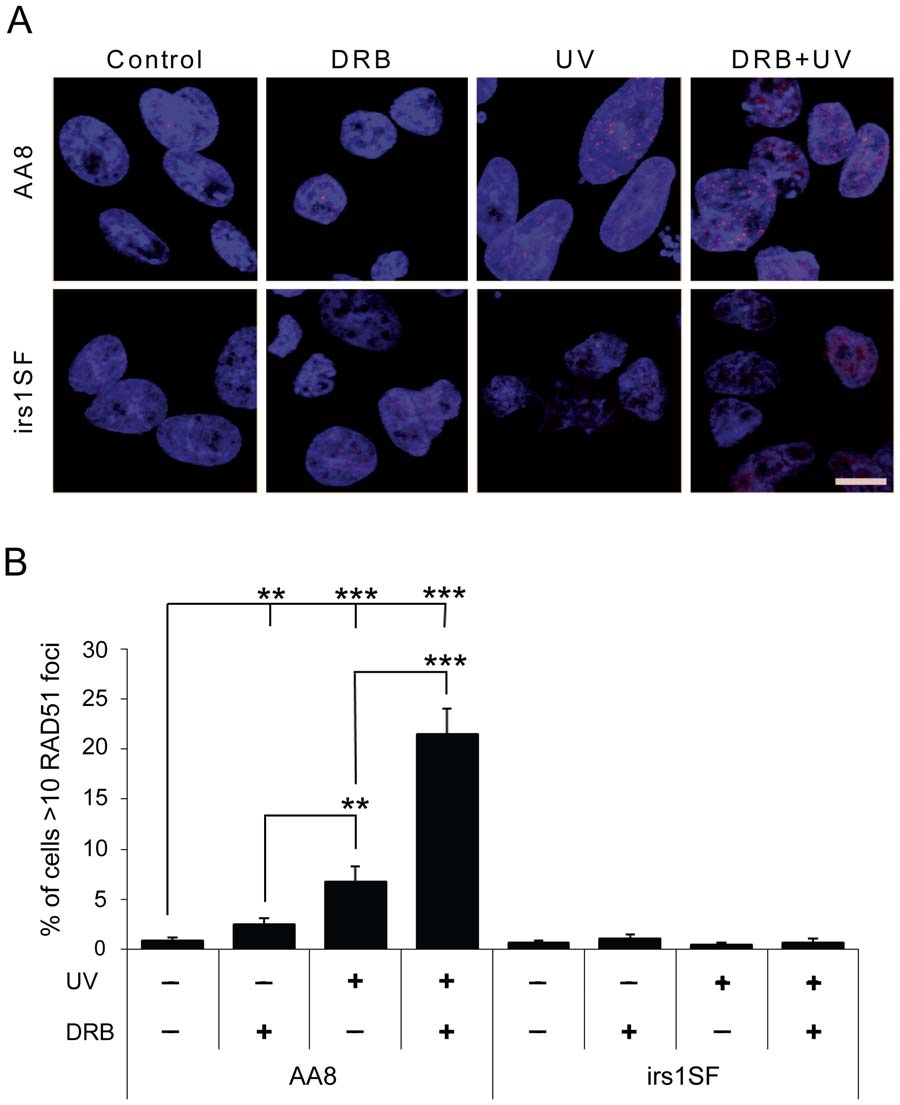
UV exposure and transcription inhibition potentiates the induction of HR in mammalian cells. AA8 and irs1SF cells were treated with 20 µM DRB for 24 h, 10 J.m^−2^ UV or both. (**A**) Confocal through focus maximum projection images showing RAD51 staining in red and DNA staining in blue. Bar 10 µm. (**B**) Quantification of RAD51 positive cells. Cells with more than 10 bright foci were considered positive. The means and S.E. (bars) of six experiments with 200–400 cells counted for each experiment are shown. Values marked with asterisks are significantly different from control in T -test (**P<0.01, ***P<0.001).

It has previously been shown that UV can induce HR [33], [34]. Here, we wanted to test if the formation of UV-induced RAD51 foci is affected by DRB treatment. In agreement with the observed increase in DNA damage, we found a synergistic increase in RAD51 foci formation in AA8 cells after combined UV and DRB treatment (Figure 5). As expected the HR defective irs1SF cells were unable to form RAD51 foci following either treatment. Similar results were observed in AA8 cells after treatment with UV and flavopiridol (Figure S3).

## Discussion

Here, we report that transcription inhibition is related to increased UV-induced toxicity. The UV sensitising effect of DRB seems to be related to an increase in UV-associated DNA damage. It has previously been found that the mutation spectrum induced by UV damage produces deletions only when associated with transcription [35], [36]. The occurrence of transcription-dependent deletions strongly suggests that DSBs are formed. In support of this, it has been reported that transcription-dependent UV-induced strand breaks form in the G1 phase of NER defective cells [37]. Here, we see γH2AX foci, a common marker of DSBs, in approximately 80% of cells, which is more than the 52% of cells normally present in the S/G2/M phases of the cell cycle (Figure 2). Hence, it is likely that some of the γH2AX foci are formed in G1 cells. However, UV-induced γH2AX foci are also formed in G1 cells in the absence of DSBs [38]. Our experiments show increased levels of 53BP1 foci, but in somewhat fewer cells as compared to the γH2AX foci, suggesting that DSB formation may be restricted to only a subset of cells. Interestingly, we see higher levels of 53BP1 foci in cells deficient in HR (Figure 4B). This is an indication that recombinational repair is involved in the removal of UV-induced DSBs, especially after combined treatment with DRB. Furthermore, we find an increase in RAD51 foci in AA8 cells, which is a likely result of initiation of recombinational repair. This repair is triggered by a UV induced DNA lesion and potentiated by DRB.

HR defective cells that are unable to repair HR-inducing lesions are normally very sensitive to agents causing such lesions. Surprisingly, we find that DRB has no UV-sensitising effect in HR defective cells, despite the fact that these cells contain more 53BP1 foci and are unable to repair the UV/DRB lesion, as observed by their inability to form RAD51 foci. In contrast, DRB efficiently sensitises NER defective UV5 or wild type AA8 cells to UV damage, despite AA8 cells forming RAD51 foci, an indication of ongoing repair.

There is a possibility that our observations are due to off-target effects of DRB and so to address this we used other transcription inhibitors to see if they too are UV sensitisers. UV sensitisation was observed in AA8 cells after addition of actinomycin D, α-amanitin or flavopiridol, but not in the HR defective irs1SF cells. Actinomycin D and α-amanitin are general inhibitors of transcription, whereas flavopiridol targets the same step of transcription as DRB and is shown to inhibit transcription *in vivo* [39]. These data suggest that the effect of DRB is related to inhibition of transcription.

Since we see increased levels of DSBs in irs1SF cells in combination with downregulation of RAD51-mediated HR repair, we would expect that DRB following UV treatment would have a stronger sensitising effect on HR defective cells in comparison to the wild type cells. Strangely, we observe the opposite effect, i.e. that HR defective cells are less sensitive to the DRB treatment. One explanation for these unusual results would be that initiation of HR (formation of RAD51 foci) without the ability to complete the process could generate a more toxic lesion capable of killing the cell. Thus, HR defective cells unable to initiate HR (RAD51 foci) would be spared, while wild type cells would die.

If this is the case, we can speculate on a model to explain such an event. Mitotic HR relies strictly on the sister chromatid as a substrate for HR, which is present after replication [27], [28]. UV-induced DSBs could form in different phases of the cell cycle, also when a sister chromatid is absent. This is supported by the high levels of γH2AX foci seen in >80% of cells, exceeding the number of cells present in the S phase of the cell cycle in an unsynchronised population; albeit not supported by the 53BP1 foci observed. The fate of a DSB will be different depending on the phase of the cell cycle. If a DSB occurs when cells are in the G1 phase of the cell cycle, there will be no resection of the DNA ends, which will result in repair by non-homologous end joining (NHEJ). This is supported by a decrease in the DRB-induced UV sensitisation in G1, observed in HR proficient cells. Complete abolition of DRB-induced UV sensitisation was not observed, which may be a result of incomplete synchronisation.

However, when cells have started replication, the activity of CDKs is high, which results in CtIP-mediated DNA end resection and onset of HR [40], [41], [42]. Normally, DSBs occurring during early S phase are associated with replication and would trigger HR at replication forks, occurring adjacent to a sister chromatid. Transcription-associated UV-induced DSBs would be uncoupled from replication and could occur far away from a replication fork. If the cell is in early S phase it would initiate end resection and loading of RAD51, unaware that no sister chromatid is present. It would than be unable to complete HR repair, which would result in futile HR repair, eventually leading to cell death.

According to this model, we would be able to explain the lack of UV-sensitisation by DRB in HR defective irs1SF cells as these would not initiate HR, but instead use NHEJ for quick repair. Wild type or NER defective cells would initiate resection and RAD51 foci formation, but would then be unable to complete HR, eventually leading to death.

In summary, we find that increased levels of UV-induced DSBs upon transcription inhibition trigger RAD51 foci formation and are toxic to wild type but not HR defective cells. We speculate that a potential mechanism to explain the lack of sensitivity to the increased amount of DSBs may be the onset of futile HR in wild type cells.

## Supporting Information

Figure S1 **The transcription inhibitors actinomycin D, α-amanitin and flavopiridol, similarly to DRB, sensitise HR-proficient, but not HR-deficient cells after treatment with UV.** Clonogenic survival assay was performed in AA8 (**A**) and irs1SF (**B**) cell lines. Cells were treated or not with 10 J.m^-2^ UV and incubated for 24 h in the presence of the transcription inhibitors actinomycin D, α-amanitin or flavopiridol in the respective concentrations. The number of colonies in non-irradiated plates for each treatment is normalised to 1 and the number of colonies in corresponding UV-irradiated plates is presented as a relative fraction of this normalised control. The histograms depict the mean and standard deviation of at least two independent experiments. The asterisks indicate statistically significant difference from control in T test (*P<0.1, ***P<0.001).
(TIF)

Figure S2 **UV dose, which gives 50% clonogenic survival in AA8, UV5 and irs1SF in the presence and absence of DRB.** The histogram shows data calculated from the experiments shown in Figure 1 and Figure 2. The bars represent doses of UV, which give 50% clonogenic survival for AA8, UV5 and irs1SF, in the presence or absence of DRB. For each cell line, the comparison was made for exponentially growing and serum starved cells. The bars depict the mean and standard deviation of at least three independent experiments. Values marked with asterisks are significantly different in T test (**P<0.01, ***P<0.001).
(TIF)

Figure S3 **The effect of flavopiridol on the RAD51 foci formation after UV treatment.** AA8 cells were treated with 10 nM flavopiridol for 24 h, 10 J.m^-2^ UV or both. The cells were fixed and stained for presence of RAD51 foci. The percentage of RAD51 positive cells was quantified. Cells with more than 10 bright foci were considered positive. The means and S.E (bars) of three experiments with 200 - 300 cells counted for each experiment are shown. Values marked with asterisks are significantly different in T test (*P<0.1, **P<0.01, ***P<0.001).
(TIF)
