# Supplementary figures and images for: Transcription Inhibition by DRB Potentiates Recombinational Repair of UV Lesions in Mammalian Cells
Source: PLoS One. 2011 May 5;6(5):e19492. doi: 10.1371/journal.pone.0019492 (PMC3088672; doi:10.1371/journal.pone.0019492)

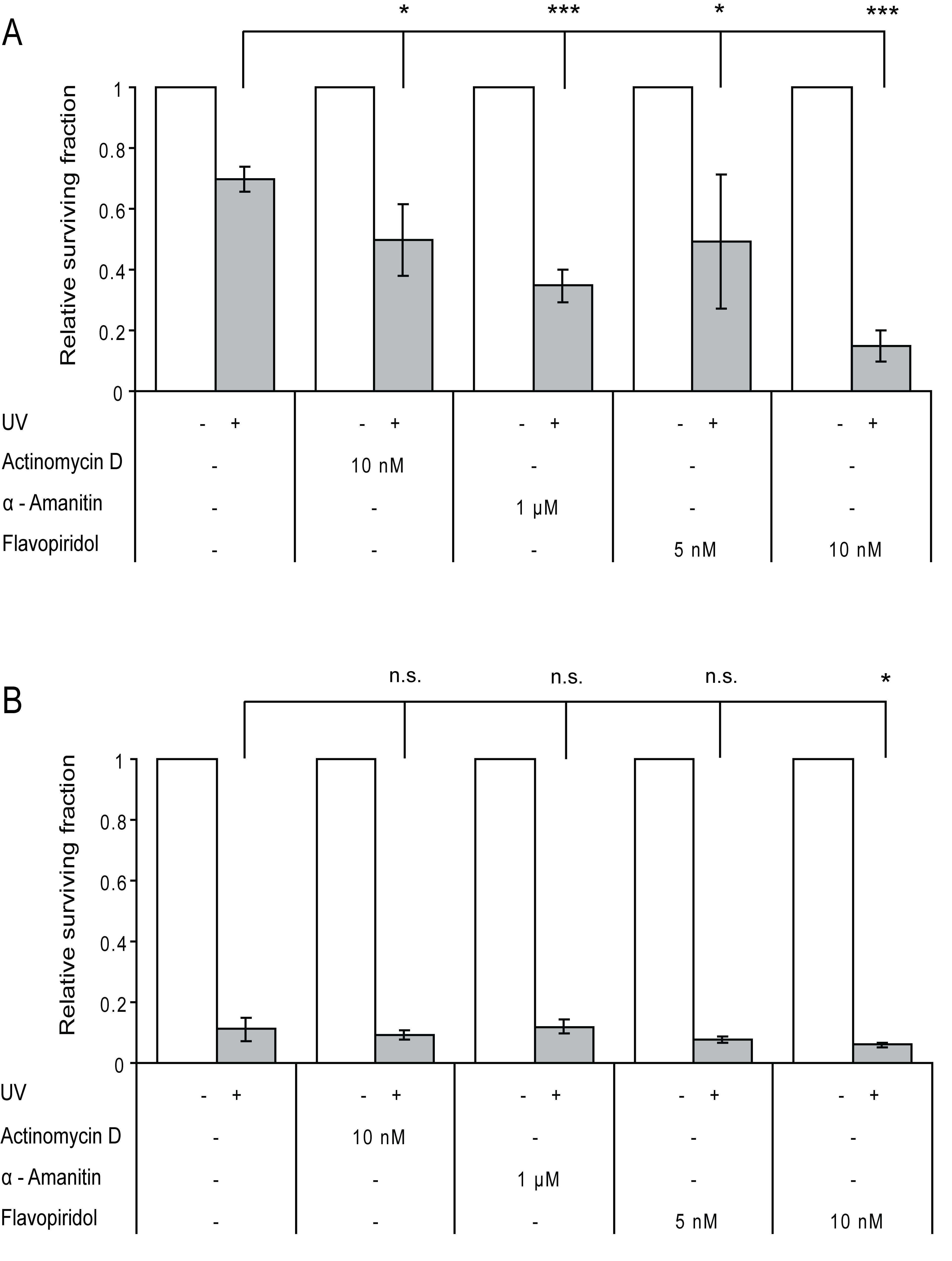

Supplement: Figure S1 — The transcription inhibitors actinomycin D, α-amanitin and flavopiridol, similarly to DRB, sensitise HR-proficient, but not HR-deficient cells after treatment with UV. Clonogenic survival assay was performed in AA8 (A) and irs1SF (B) cell lines. Cells were treated or not with 10 J.m-2 UV and incubated for 24 h in the presence of the transcription inhibitors actinomycin D, α-amanitin or flavopiridol in the respective concentrations. The number of colonies in non-irradiated plates for each treatment is normalised to 1 and the number of colonies in corresponding UV-irradiated plates is presented as a relative fraction of this normalised control. The histograms depict the mean and standard deviation of at least two independent experiments. The asterisks indicate statistically significant difference from control in T test (*P<0.1, ***P<0.001). (TIF) [file pone.0019492.s001.tif]

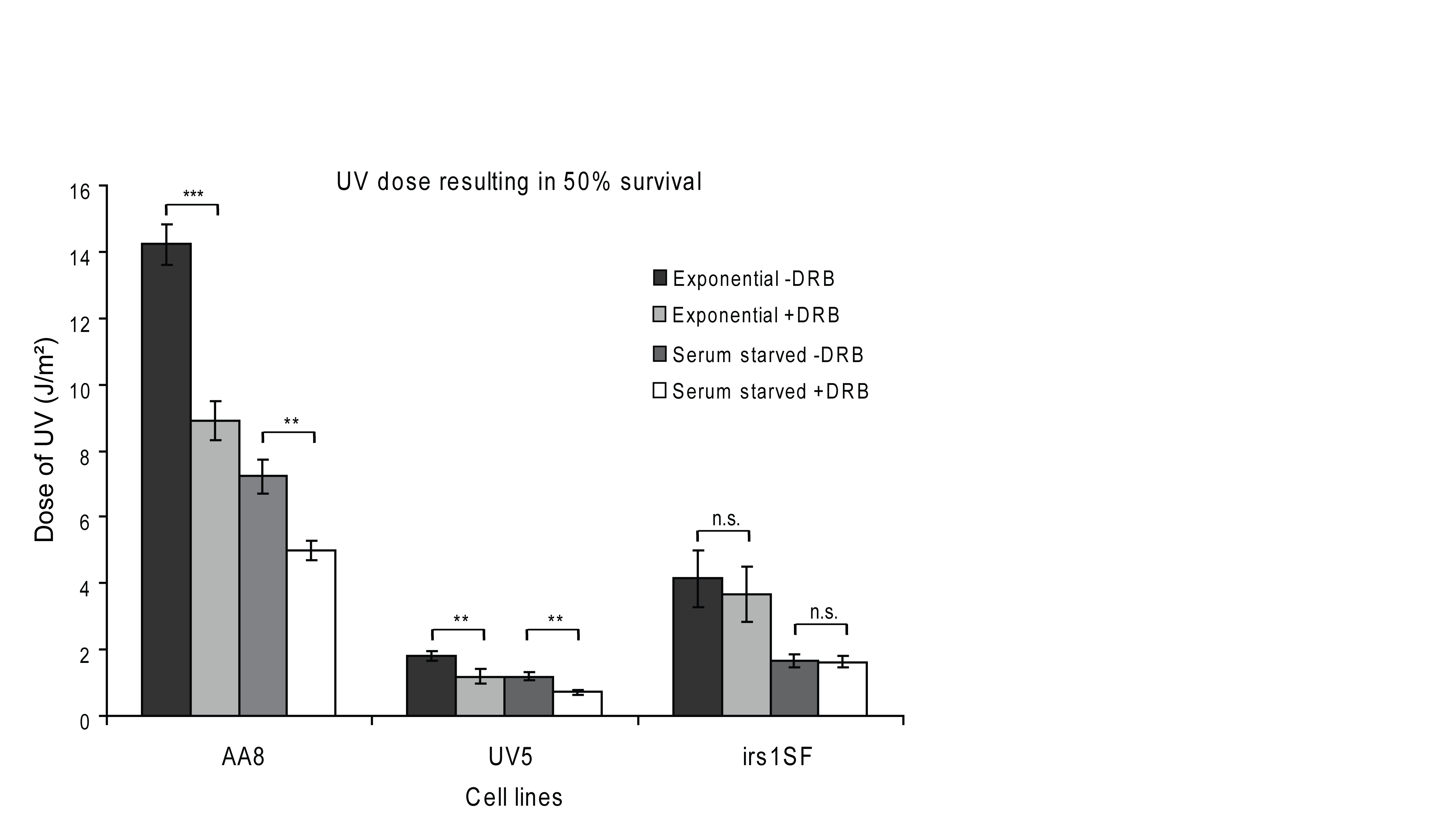

Supplement: Figure S2 — UV dose, which gives 50% clonogenic survival in AA8, UV5 and irs1SF in the presence and absence of DRB. The histogram shows data calculated from the experiments shown in Figure 1 and Figure 2. The bars represent doses of UV, which give 50% clonogenic survival for AA8, UV5 and irs1SF, in the presence or absence of DRB. For each cell line, the comparison was made for exponentially growing and serum starved cells. The bars depict the mean and standard deviation of at least three independent experiments. Values marked with asterisks are significantly different in T test (**P<0.01, ***P<0.001). (TIF) [file pone.0019492.s002.tif]

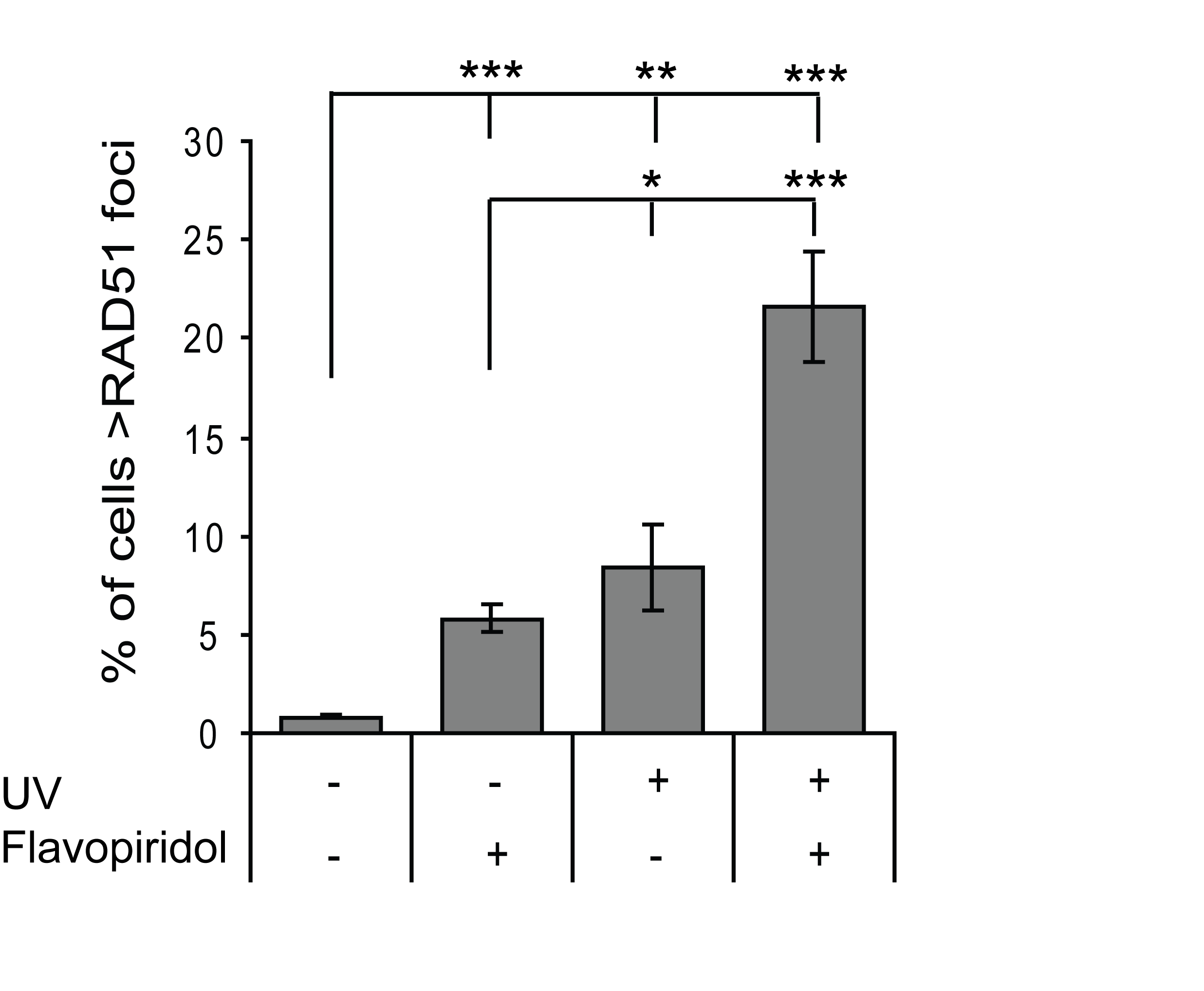

Supplement: Figure S3 — The effect of flavopiridol on the RAD51 foci formation after UV treatment. AA8 cells were treated with 10 nM flavopiridol for 24 h, 10 J.m-2 UV or both. The cells were fixed and stained for presence of RAD51 foci. The percentage of RAD51 positive cells was quantified. Cells with more than 10 bright foci were considered positive. The means and S.E (bars) of three experiments with 200 - 300 cells counted for each experiment are shown. Values marked with asterisks are significantly different in T test (*P<0.1, **P<0.01, ***P<0.001). (TIF) [file pone.0019492.s003.tif]
